# Expression Profiles and Ontology Analysis of Circular RNAs in a Mouse Model of Myocardial Ischemia/Reperfusion Injury

**DOI:** 10.1155/2020/2346369

**Published:** 2020-05-20

**Authors:** Zhaoqing Sun, Tongtong Yu, Yundi Jiao, Dongxu He, Jiake Wu, Weili Duan, Zhijun Sun

**Affiliations:** Department of Cardiology, Shengjing Hospital of China Medical University, Shenyang 110022, China

## Abstract

Circular RNAs (circRNAs) play important roles in cellular physiology. The association between circRNAs and myocardial ischemia/reperfusion (I/R) injury remains largely unknown. The aim of this study was to test the effects of myocardial I/R circRNA expression and explore the potential roles of these circRNAs. CircRNAs were screened by high-throughput sequencing, and the expression of dysregulated circRNAs was further validated using quantitative real-time polymerase chain reaction. Nineteen upregulated and 20 downregulated circRNAs were identified. Gene Ontology analysis indicated that the dysregulated transcripts were associated with fundamental pathophysiologic processes. Kyoto Encyclopedia of Genes and Genomes pathway analysis showed significant changes in adherens junction, the HIF-1 signaling pathway, the cell cycle, and the FoxO signaling pathway which have a close relationship with myocardial I/R injury. The circRNA-miRNA analysis demonstrated the broad potential of the differentially expressed circRNAs to regulate target genes by acting on the miRNAs. This study provides a foundation for understanding the roles and mechanisms of circRNAs in myocardial I/R injury.

## 1. Introduction

Acute myocardial infarction (AMI) is the result of a precipitous decline in coronary artery blood flow. This condition seriously threatens human health. The restoration of blood flow can significantly improve prognosis. However, many studies have indicated that myocardial reperfusion can also induce additional damage to the myocardium, defined as myocardial ischemia/reperfusion (I/R) injury [1, 2]. Furthermore, clinical studies have determined that, even after receiving timely and optimal myocardial reperfusion, patients still experience a high rate of death and a high incidence of cardiac failure after AMI [3]. Myocardial I/R injury plays an important role in the process [1, 2]. Over the past 30 years, many cardioprotective strategies against I/R injury have been proposed. However, practice shows that the current effects are not ideal [4]. We need to further study the pathophysiological process of myocardial ischemia reperfusion and explore new therapeutic targets. Circular RNAs (circRNAs) are a large family of noncoding RNAs. These molecules are characterized by the formation of a covalently closed loop structure through joining the 3′ end of the RNAs to the 5′ end [5, 6]. Recently, circRNAs have become an area of increased research focus. Increasing research has confirmed the association between circRNAs and physiological and pathological development in many organisms. As microRNA (miRNA) sponges, circRNAs can interact with RNA binding proteins (RBPs) and control alternative splicing and parental gene expression. CircRNAs play an important regulatory role at the transcriptional and posttranscriptional levels [7–9]. An increasing number of studies have found that circRNAs are also involved in many kinds of cardiovascular diseases [10, 11]. However, the signature of circRNA expression and the possible roles of circRNAs in myocardial I/R injury have received relatively little attention. In this study, we tested the effects of myocardial I/R injury on circRNA expression and explored the potential roles of these circRNAs at the early stage of reperfusion.

## 2. Materials and Methods

### 2.1. Animal

Male C57BL/6 mice (10–12 weeks old, body weight approximately 25 g, from Beijing Huafu Kang Animal Experiment Company) were used in this study. Animal experiments were conducted following the NIH Guide for Laboratory Animals. All mice were fed with standard mouse food and water. The protocol was approved by the Experimental Animal Ethics Committee of Shengjing Hospital of China Medical University, China (Animal Experimental Ethical Inspection Protocol No. 2017PS041K).

### 2.2. Myocardial I/R Injury Protocol

Six C57BL/6 mice were randomly assigned to two groups: the I/R group and the control group (sham group). Myocardial I/R was performed as described previously (45 min of ischemia followed by 2 h of reperfusion) [12]. Briefly, C57BL/6 mice were first anesthetized with pentobarbital (100 mg/kg, intraperitoneal injection), and an additional dose could be needed during the experiment to maintain anesthesia. This procedure was followed by intubation and ventilation. The left anterior descending artery (LAD) of each mouse was ligatured with an 8–0 prolene suture to induce myocardial ischemia, which was confirmed by the changes in the ECG and the presence of epicardial cyanosis. After 45 min of ischemia, the occlusion of the left anterior coronary artery was released and reperfusion occurred which was verified by visualizing a marked epicardial hyperemic response. Animals were sacrificed at the time of reperfusion (2 h after 45 min ischemia). At autopsy, samples were collected from the infarct region as quickly as possible [13] and the samples were confirmed by visual inspection under a dissecting microscope by discoloration of the occluded distal myocardium. The sham group underwent the same surgical procedure, including thread drawing, but not ligation at the same site. We collected samples from the control group in the myocardial region of the LAD blood supply.

### 2.3. High-Throughput circRNA Sequencing

High-throughput circRNA sequencing was performed in Beijing Novogene with six samples (3 samples from the control group and 3 from the I/R group). The procedure was as follows: after extracting the total RNA, the ribosomal RNA (rRNA) was removed from the total RNA. RNA-seq libraries were constructed. Then, double-strand complementary DNA (cDNA) was synthesized. After removing second-strand cDNA, first-strand cDNA was purified for PCR amplification. Then, the samples were sequenced with Illumina HiSeq™ 2500 (Illumina) using the paired end (PE) sequencing strategy according to the Illumina protocol. The raw data were recorded. Then, sequence quality was evaluated with the quality control software FastQC and C clean reads were available. Find_circ (v1.0) and CIRI2 were used to recognize circRNAs [14, 15].

### 2.4. Bioinformatic Analyses

The differential expression of circRNAs was analyzed by DEGseq [16]. Gene Ontology (GO) analysis (http://www.geneontology.org/) was used to obtain information about the gene-related biological processes and molecular functions [17]. Kyoto Encyclopedia of Genes and Genomes (KEGG; http://www.kegg.jp) pathway analysis was also performed to provide information about the molecular interaction and reaction networks of the differentially expressed circRNAs [18].

### 2.5. Quantitative Real-Time Polymerase Chain Reaction

Quantitative real-time polymerase chain reaction (qPCR) was conducted to confirm the differential expression of circRNAs [19]. Total RNA from cardiac tissues was extracted using the TRIzol Reagent (Takara, Dalian, China). Primers were designed using Primer 5 software, and all primers spanned the distal ends of circRNAs. cDNA was synthesized using the PrimeScript™ RT Reagent Kit (Takara, Dalian, China). The qPCR analyses were performed using a TB Green™ Premix Ex Taq™ II (Takara, Dalian, China) and an ABI Q2Real-time PCR system (Applied Biosystems, Foster City, CA, USA). The relative expression of different circRNAs was calculated by the 2^−ΔΔCt^ method.

### 2.6. Statistical Methods

Data are expressed as the means ± standard deviation. Student's *t*-test was performed for comparisons between two groups. Differences with *P* < 0.05 were considered statistically significant.

## 3. Results

### 3.1. Different Expression Profiles of circRNAs

**Figure 1:**
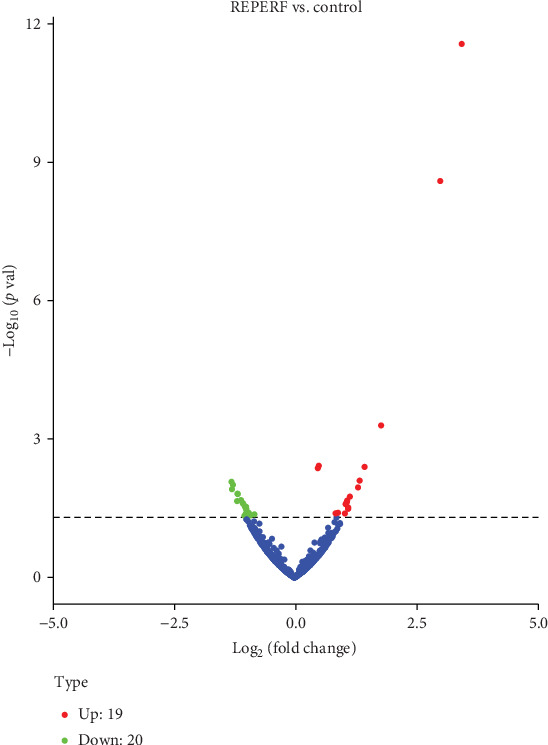
Volcano plots of the significantly differentially expressed circRNAs. CircRNAs were detected both in the I/R group and the control group. Compared with those in the control group, 39 were differentially expressed in the I/R group, among which 19 were upregulated (red dots) and 20 were downregulated (green dots). circRNAs: circular RNAs; REPERF: ischemia/reperfusion group.

**Figure 2:**
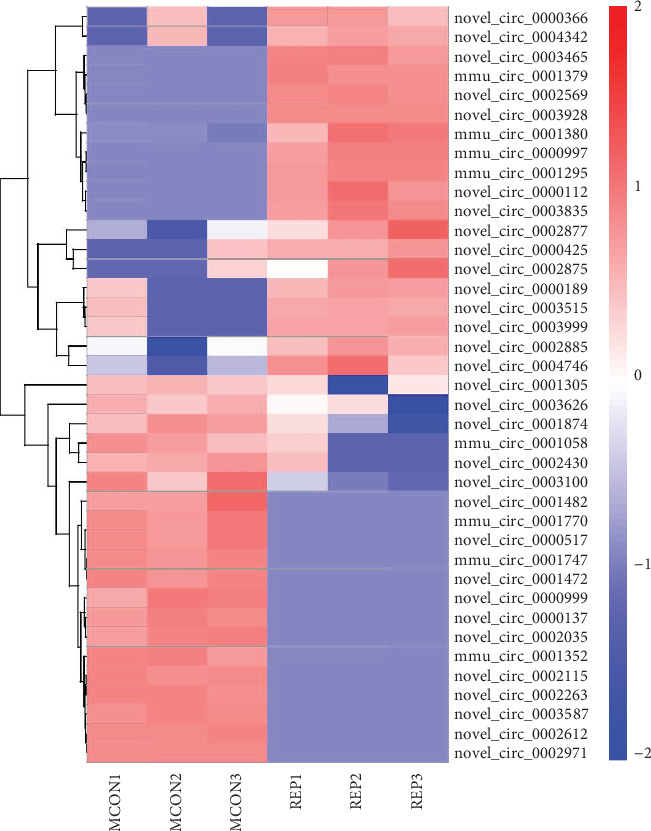
Hierarchical clustering of circRNA expression patterns between the I/R group and control group. CircRNA expression patterns between the I/R group and control group were different. Overexpressed circRNAs are presented by red rectangles. Underexpressed circRNAs are presented by blue rectangles. REP: ischemia/reperfusion group; MCON: control group; circRNA: circular RNA.

A total of 920 novel and 590 known circRNAs were detected by RNA sequencing in all samples. Among them, 39 were differentially expressed (19 up- and 20 downregulated) in the I/R group, compared with the control group. Volcano plots visualized the significantly differentially expressed circRNAs (Figure 1). Thirty-one circRNAs were identified as new circRNAs that had not been annotated in the circBase or Circ2Traits database [20, 21]. The results of hierarchical clustering suggested that the circRNA expression patterns were distinguishable between the I/R and control groups (Figure 2). All dysregulated circRNAs are listed in Table 1.

### 3.2. Validation of circRNA Expression

**Figure 3:**
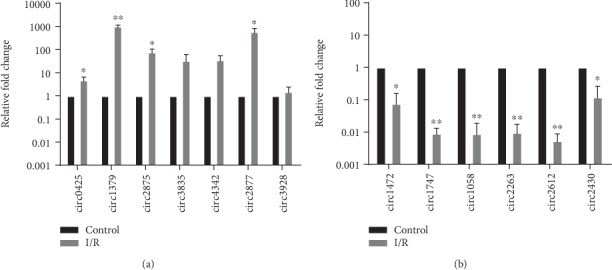
To confirm the differential expression of circRNAs between the control group and the I/R group; qPCR was conducted. ^∗^*P* < 0.05 and ^∗∗^*P* < 0.01. qPCR: quantitative real-time polymerase chain reaction; I/R group: ischemia/reperfusion group.

To verify the RNA sequencing data, we randomly selected 13 dysregulated circRNAs including 7 upregulated circRNAs (0001379, 0002875, 0002877, 0004342, 0000425, 0003835, and 0003928) and 6 downregulated circRNAs (0001747, 0001472, 0001058, 0002263, 0002430, and 0002612) for verification in samples. A general consistency was shown between the real-time PCR and sequencing analysis results. Four selected upregulated circRNAs and six selected downregulated circRNAs were confirmed (Figures 3(a) and 3(b)).

### 3.3. GO and KEGG Analyses

**Figure 4:**
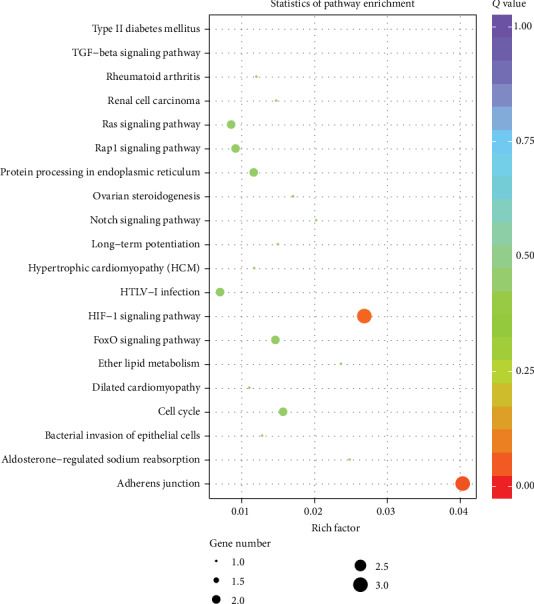
Molecular interactions among genes explored by KEGG pathway analysis (top 20 pathways). KEGG enrichment was measured by rich factor, *Q* value, and gene number. A bigger value of rich factor means better enrichment. The lower value of *Q* value means better enrichment. KEGG: Kyoto Encyclopedia of Genes and Genomes.

GO analysis of the host genes of the differentially expressed circRNAs was used to predict the circRNA functions including three domains: biological process, cellular component, and molecular function.  Table 2 shows the top 10 enriched GO terms in biological process, cellular component, and molecular function, respectively. The most significant GO functions were associated with 3-5 RNA helicase activity, RNA polymerase II transcription factor binding, condensed chromosome outer kinetochore, condensed chromosome kinetochore, mitochondrial ncRNA surveillance, and mitochondrial mRNA surveillance. Additionally, the molecular interactions among the genes were explored by KEGG pathway analysis. The results are presented in Figure 4. The host genes of the differentially expressed circRNAs were mainly associated with adherens junction, the HIF-1 signaling pathway, the cell cycle, and the FoxO signaling pathway.

### 3.4. Analysis of circRNA-miRNA Interactions

**Figure 5:**
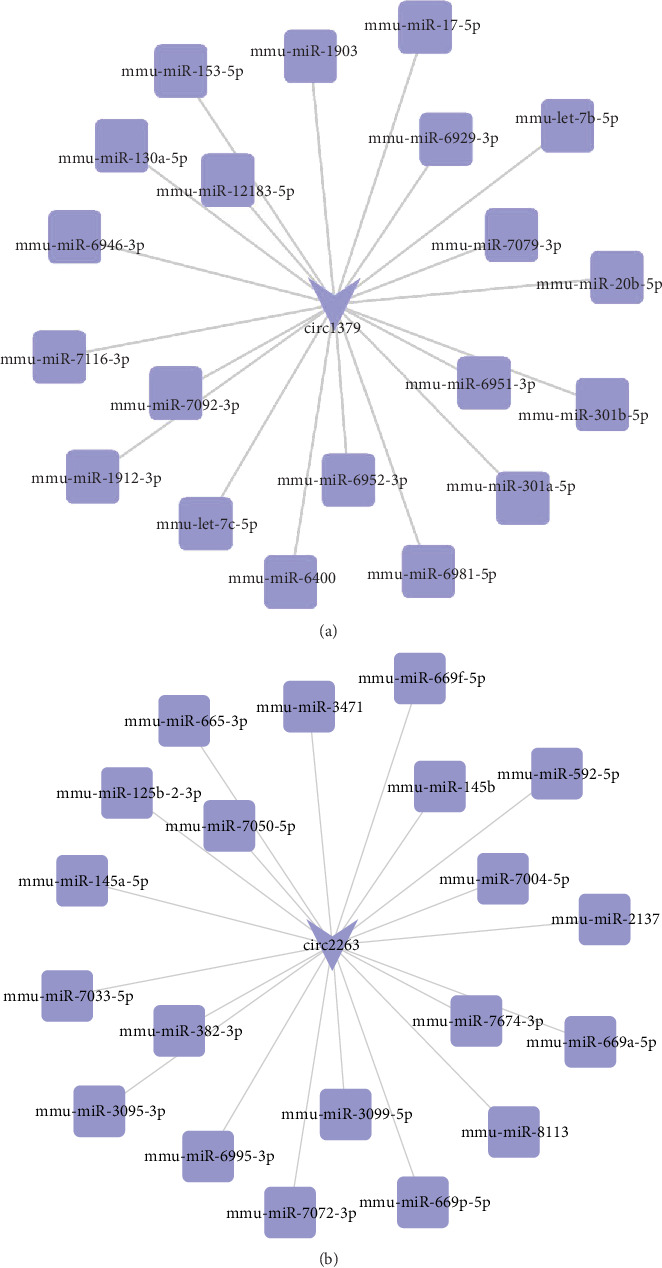
circRNA-miRNA interaction network.

It has been confirmed that circRNAs can regulate the expression of genes as miRNA sponges. Hence, we predicted the potential target miRNAs of the confirmed differentially expressed circRNAs using miRanda software. A circRNA-miRNA interaction network was constructed using Cytoscape3.4.0 software. Two confirmed circRNAs (mmu_circRNA_0001379 and mmu_circRNA_0002263) were selected, and the circRNA/microRNA interaction was predicted. The potential miRNA targets of mmu_circRNA_0001379 include miR-153-5p, miR-130a-5p, and miR-17-5p (top 20, Figure 5(a)). For mmu_circRNA_0002263, the potential miRNA targets include miR-145a-5p, miR-145b, and miR-669p-5p (top 20, Figure 5(b)). It has been shown that miRNA-17-5p promoted cardiomyocyte apoptosis induced by oxidative stress via targeting Stat3 in an animal model of I/R injury [22], while the function of the most potential miRNAs on the heart are far from clear.

## 4. Discussion

In this study, we exhibited the specific circRNA expression profile in I/R heart tissues at the early stage of reperfusion and provided some potential targets and pathways of the circRNA involved in I/R injury. These results provide important clues to reveal the role of circRNA in the pathologic process of cardiac I/R.

Based on the high-throughput sequencing data, we found significant upregulation of 19 circRNAs and downregulation of 20 circRNAs in the I/R group compared to the control group. Of them, 31 circRNAs were identified as new circRNAs that had not been annotated in the circBase database. Garikipati et al. performed circRNA microarray analysis using RNA isolated from sham or MI mouse hearts at day 3 post-MI. They found that 82 circRNAs were differentially expressed, including 41 upregulated and 41 downregulated [23]. Ge et al. found that a total of 185 significantly differentially expressed circRNAs were identified in EVs from the murine heart post-I/R injury, such as mmu-circ008351 and mmu-circ001007 [24]. Wu et al. applied a microarray assay to examine the transcriptome of circRNAs using a postmyocardial infarction model of heart failure in mice. They found that a total of 63 circRNAs were differential expressed, consisting of 29 upregulated circRNAs and 34 downregulated circRNAs [25]. We did not detect these circRNAs in our study, maybe because we used an I/R model at the early stage of reperfusion and one which is different from the model used in other studies.

To gain insight into the potential roles of the differentially expressed circRNAs in the infarct region, we performed GO and pathway analysis to predict the biological functions and mechanisms of the correlated targets. The most significant GO functions were associated with 3-5 RNA helicase activity, RNA polymerase II transcription factor binding, condensed chromosome outer kinetochore, condensed chromosome kinetochore, mitochondrial ncRNA surveillance, and mitochondrial mRNA surveillance. In order to get more credible biological functions, we used KEGG pathway analysis and found that the dysregulated transcripts were associated with adherens junction, the HIF-1 signaling pathway, the cell cycle, and the FoxO signaling pathway. Tansey et al. studied the reduction and redistribution of gap and adherens junction proteins after ischemia and reperfusion and they found that there are significant alterations in the structural integrity of the myocardium as well as gap and adherens junction protein expression with increasing global ischemia time [26]. Liu et al. found that microRNA-138 attenuates myocardial ischemia reperfusion injury through inhibiting mitochondria-mediated apoptosis by targeting HIF-1*α* [13]. Liem et al. found that the cell cycle proteins, Rb and Cdk2, are critical regulators of cardiac myocyte injury in vivo and support a cardioprotective role for Rb during I/R injury [27]. Qi et al. found that the FoxO3a pathway was involved in the I/R injury of cardiac microvascular endothelial cells (CMECs) at least in part through the regulation of cell cycle arrest and apoptosis [28]. Therefore, the four signaling pathways are involved with ischemia/reperfusion injury. Further researches are still needed to confirm these findings.

Large amounts of circRNAs have recently been discovered and represent a new special class of endogenous noncoding RNA. Recent studies have revealed that circRNAs are an abundant, stable, diverse, and conserved class of RNA molecules. Moreover, circRNAs can function as miRNA sponges or regulate parent gene expression to affect disease initiation and progression [11]. To date, many studies have reported associations between circRNAs and cardiovascular disease [10, 11]. The overexpression of circRNA CDR1as in hypoxic cardiomyocytes and mice with myocardial infarction has been found. CircRNA CDR1as could enhance the expression of the miR-7 target genes PARP and SP1 and increase the cardiac infarct size [29]. Recently, more studies revealed that circRNAs can function mainly as competing endogenous RNAs or miRNA sponges [11]. In our study, we found many dysregulated circRNAs in the heart tissue during I/R injury and we predicted the circRNA/microRNA interaction with miRanda software. We found that upregulated mmu_circRNA_0001379 can tightly bind miR-17-5p and is a potential sponge of miR-17-5p. A previous study also showed that miR-17-5p promoted cardiomyocyte apoptosis induced by oxidative stress via targeting Stat3 in both the animal model of I/R injury and the cellular model of cardiomyocyte injury induced by oxidative stress [22]. mmu_circRNA_0001379 may represent a new type of mediator of cardiac I/R injury. We infer that circRNA0001379 may act as an efficient miR-17-5p sponge and then affect the expression of downstream mRNAs. Due to the limited known function of circRNAs and miRNAs, many circRNA/microRNA interactions should be analyzed in the future.

However, there are some limitations in our study. First, the samples we selected are comparatively limited. In this study, we have only explored 3 control samples and 3 reperfusion samples for high-throughput sequencing analysis. More samples are needed to verify the results in future research. Second, the length of reperfusion time for sample collection was short and lasted only 2 h. Therefore, whether the expression of the circRNAs would show the same change after a longer period of ischemia is unknown. More studies in the future will be conducted for further exploration.

## 5. Conclusions

In conclusion, we initiated an exploratory analysis of the expression of circRNAs at the early stage of reperfusion in the heart and investigated some of their potential roles and mechanisms. The identified circRNAs might serve as novel targets with further potential to prevent myocardial I/R injury.

## Figures and Tables

**Table 1:** Dysregulated circRNAs in the I/R group compared with those in the control group.

CircRNA ID	Log_2_FC	*P* value	Regulation	Chromosome	Gene name
mmu_circ_0001058	-1.289	0.012	Down	2	Bub1b
novel_circ_0001305	-1.002	0.040	Down	4	Rere
novel_circ_0001874	-0.997	0.029	Down	X	6720401G13Rik
novel_circ_0002430	-1.184	0.022	Down	10	Adarb1
novel_circ_0003100	-0.827	0.042	Down	11	Sec24a
novel_circ_0003626	-1.032	0.046	Down	12	Foxn3
mmu_circ_0001352	-1.269	0.010	Down	5	Apbb2
mmu_circ_0001747	-1.102	0.021	Down	9	Chordc1
mmu_circ_0001770	-0.946	0.040	Down	9	Pafah1b2
novel_circ_0000137	-0.912	0.045	Down	1	A330023F24Rik
novel_circ_0000517	-0.946	0.040	Down	14	Erc2
novel_circ_0000999	-1.299	0.008	Down	2	Dnm1
novel_circ_0001472	-1.170	0.015	Down	6	Ezh2
novel_circ_0001482	-1.054	0.025	Down	6	Prdm5
novel_circ_0002035	-1.020	0.029	Down	10	Fam19a2
novel_circ_0002115	-0.995	0.033	Down	10	Ahi1
novel_circ_0002263	-1.070	0.024	Down	10	Ascc3
novel_circ_0002612	-1.175	0.015	Down	10	None
novel_circ_0002971	-0.919	0.044	Down	11	Psme4
novel_circ_0003587	-0.995	0.033	Down	11	Npepps
mmu_circ_0001379	3.448	0.000	Up	5	Zfp644
novel_circ_0002569	3.005	0.000	Up	10	Rmst
mmu_circ_0001295	1.786	0.001	Up	4	Rere
novel_circ_0002875	0.481	0.004	Up	11	Vps54
novel_circ_0002877	0.501	0.004	Up	11	Ugp2
novel_circ_0004342	1.344	0.008	Up	12	None
novel_circ_0000425	1.311	0.011	Up	12	Esyt2
mmu_circ_0001380	1.086	0.021	Up	5	Zfp644
novel_circ_0000366	1.104	0.032	Up	12	Dgkb
novel_circ_0002885	0.847	0.040	Up	11	Ehbp1
novel_circ_0003515	1.108	0.032	Up	11	Trim37
novel_circ_0004746	0.898	0.039	Up	13	Hiatl1
mmu_circ_0000997	1.081	0.023	Up	2	Prrc2b
novel_circ_0000112	1.054	0.026	Up	1	Gpr161
novel_circ_0000189	1.039	0.041	Up	10	Zfr2
novel_circ_0003465	1.068	0.024	Up	11	Synrg
novel_circ_0003835	1.142	0.018	Up	12	Asap2
novel_circ_0003928	1.446	0.004	Up	12	None
novel_circ_0003999	1.101	0.030	Up	12	Itsn2

circRNAs: circular RNAs; I/R group: ischemia/reperfusion group.

**Table 2:** Top 10 enriched GO terms in molecular function, cellular component, and biological process.

Term	Term type	*P* value
3-5 RNA helicase activity	Molecular function	0.0010346
RNA polymerase II transcription factor binding transcription factor activity involved in negative regulation of transcription	Molecular function	0.0015731
Zinc ion binding	Molecular function	0.0015795
Beta-N-acetylglucosaminidase activity	Molecular function	0.0021346
VEGF-A-activated receptor activity	Molecular function	0.0038123
VEGF-B-activated receptor activity	Molecular function	0.0038123
Placental growth factor-activated receptor activity	Molecular function	0.0038123
Histone acetyltransferase activity	Molecular function	0.0050248
Transition metal ion binding	Molecular function	0.0052732
Insulin-activated receptor activity	Molecular function	0.0072851
Condensed chromosome outer kinetochore	Cellular component	0.00016441
Condensed chromosome kinetochore	Cellular component	0.00053713
Condensed chromosome, centromeric region	Cellular component	0.00098589
Mitochondrial degradosome	Cellular component	0.0029943
DBIRD complex	Cellular component	0.0032617
Intracellular organelle lumen	Cellular component	0.0054793
Organelle lumen	Cellular component	0.0055775
Membrane-enclosed lumen	Cellular component	0.0061175
Insulin receptor complex	Cellular component	0.007035
Myosin complex	Cellular component	0.0087096
Mitochondrial ncRNA surveillance	Biological process	0.0010346
Mitochondrial mRNA surveillance	Biological process	0.0010346
Cytoplasmic RNA surveillance	Biological process	0.0010346
Mitochondrial RNA surveillance	Biological process	0.0010346
Regulation of rho-dependent protein serine/threonine kinase activity	Biological process	0.0011299
Negative regulation of rho-dependent protein serine/threonine kinase activity	Biological process	0.0011299
Cellular response to heat	Biological process	0.0019831
Regulation of cardiac muscle adaptation	Biological process	0.0021346
Negative regulation of cardiac muscle adaptation	Biological process	0.0021346
Regulation of cellular response to heat	Biological process	0.0023189

GO: Gene Ontology.

## Data Availability

The data used to support the findings of this study are available from the corresponding author upon request.
