# Erratum to “Expression Profiles and Ontology Analysis of Circular RNAs in a Mouse Model of Myocardial Ischemia/Reperfusion Injury”

**DOI:** 10.1155/2020/2097954

**Published:** 2020-11-23

**Authors:** Zhaoqing Sun, Tongtong Yu, Yundi Jiao, Dongxu He, Jiake Wu, Weili Duan, Zhijun Sun

**Affiliations:** Department of Cardiology, Shengjing Hospital of China Medical University, Shenyang 110022, China

In the article titled “Expression Profiles and Ontology Analysis of Circular RNAs in a Mouse Model of Myocardial Ischemia/Reperfusion Injury” [1], there was an error in the corresponding author list. The corrected corresponding author details are shown above. This mistake was introduced during the production of the article and the publisher apologizes for this error.
